# Changes in selected exerkines concentration post folk-dance training are accompanied by glucose homeostasis and physical performance improvement in older adults

**DOI:** 10.1038/s41598-023-35583-w

**Published:** 2023-05-26

**Authors:** Ewa Aleksandra Rodziewicz-Flis, Małgorzata Kawa, Jan Jacek Kaczor, Marzena Szaro-Truchan, Damian Józef Flis, Giovanni Lombardi, Ewa Ziemann

**Affiliations:** 1 Department of Basic Physiotherapy, Gdansk University of Physical Education and Sport, Gdansk, Poland; 2 Department of Animal and Human Physiology, University of Gdansk, Gdansk, Poland; 3 Department of Pharmaceutical Pathophysiology, Medical University of Gdansk, Gdansk, Poland; 4 Laboratory of Experimental Biochemistry and Molecular Biology, IRCCS Istituto Ortopedico Galeazzi, Milan, Italy; 5 Department of Athletics, Strength and Conditioning, Poznan University of Physical Education, Poznan, Poland

**Keywords:** Disease prevention, Geriatrics, Biochemistry, Cytokines, Physiology, Ageing, Metabolism

## Abstract

The study aimed to evaluate the impact of selected exerkines concentration induced by folk-dance and balance training on physical performance, insulin resistance, and blood pressure in older adults. Participants (n = 41, age 71.3 ± 5.5 years) were randomly assigned to folk-dance (DG), balance training (BG), or control group (CG). The training was performed 3 times a week for 12 weeks. Physical performance tests—time up and go (TUG) and 6-min walk test (6MWT), blood pressure, insulin resistance, and selected proteins induced by exercise (exerkines) were assessed at baseline and post-exercise intervention. Significant improvement in TUG (p = 0.006 for BG and 0.039 for DG) and 6MWT tests (in BG and DG p = 0.001), reduction of systolic blood pressure (p = 0.001 for BG and 0.003 for DG), and diastolic blood pressure (for BG; p = 0.001) were registered post-intervention. These positive changes were accompanied by the drop in brain-derived neurotrophic factor (p = 0.002 for BG and 0.002 for DG), the increase of irisin concentration (p = 0.029 for BG and 0.022 for DG) in both groups, and DG the amelioration of insulin resistance indicators (HOMA-IR p = 0.023 and QUICKI p = 0.035). Folk-dance training significantly reduced the c-terminal agrin fragment (CAF; p = 0.024). Obtained data indicated that both training programs effectively improved physical performance and blood pressure, accompanied by changes in selected exerkines. Still, folk-dance had enhanced insulin sensitivity.

## Introduction

Reduced daily physical activity, which has been further pushed by the SARS-CoV-2 pandemic period is observed both in young and older adults ^1,2^. The sedentary lifestyle increased risk of developing many chronic diseases and pathological states such as obesity, insulin resistance, chronic inflammation, type 2 diabetes, and cardiovascular disease (CVD)^3^. these consequences are closely related to sarcopenia and physical performance deterioration^4^ In addition, this series of problems mainly affect the elderly because, on the one hand, aging triggers and accelerates these pathological states and, on the other hand, by contextualizing this phenomenon into the current time, aged people are the group that mainly suffers from the "side effects" of the pandemics^5,6^. Thus, searching for potential effective methods that may ameliorate a condition of insulin resistance and counteract the development of type 2 diabetes seems to be essential in preventing dementia or sarcopenia, leading at the same time to healthy aging.

Physical exercise can convey a protective effect against aging-related inflammation, releasing proteins named myokines into the bloodstream during muscle contractions^7,8^. Although, the more than 600 myokines is known, biological function has been described for only 5% of them^9^. Available data have documented the role of selected myokines in lipid and glucose metabolism, or muscle hypertrophy. Therefore, they may be useful biomarkers in monitoring the effectiveness of regular physical activity. Myokines can interact between distant structures and stimulate other organs to release proteins in response to exercise, which are named exerkines^10^. Among several exerkines: brain-derived neurotrophic factor (BDNF)^10^ and irisin^11^ are known as promising exercise-dependent mediators with health-promoting effects. BDNF's primary function is to regulate neurogenesis and cognitive functions, but it also affects lipid and glucose metabolism^12,13^ in both the central nervous system and the periphery. A recently published study identified a new β-cell signalling pathway regulating insulin secretion in mammals and demonstrated the role of BDNF as a soluble ligand activating TrkB.T1 receptor expressed by β-cells of the pancreas^14^. Most published studies indicated that circulating BDNF levels increase in response to single exercise bouts and regular physical activity^15,16^. Moreover, animal models revealed that irisin may cross the blood–brain barrier and enter the central nervous system, where it can stimulate the expression of BDNF^17^. It has also been suggested that irisin plays a crucial role in regulating glucose and lipid metabolism in adipose tissue and skeletal muscles^18^.

Myostatin together with C-terminal agrin fragment (CAF) control skeletal muscle mass loss and have a particular meaning in counteracting the development of muscle atrophy and sarcopenia^19–22^. Myostatin is responsible for skeletal muscle wasting by inhibiting protein synthesis and regenerative processes, as occurs in aging. In turn, CAF through inducing muscle wasting is considered as a clinical biomarker for neuromuscular junction (NMJ) degeneration ^23^. Its increased circulating concentration, secondary to enhanced agrin cleavage, reflects progressive myofibers denervation that finally contributes to muscle dysfunction and atrophy^24^.. Although it is known that physical training prevents motor neuron and NMJ degeneration, muscle fiber denervation, and loss of motor units, along with the modification of circulating myostatin and CAF, the conclusions are not consistent^25–27^.

One of the most popular forms of physical activity among the elderly is Nordic Walking (NW), still there is need to search for the most effective training methods and potential mechanisms leading to healthy aging. Nowadays, more attention is paid to dance-based interventions, that comprised several different stimuli, what may lead to improvement in physical fitness, balance, muscle strength and extended neuroplasticity in healthy older adults^28,29^. Thus, the aim of this study was to assess the effectiveness of two training programs—folk-dance and balance on older adults’ physical performance, insulin resistance, blood pressure, inflammation, and lipid profile in conjunction with selected exerkines changes. We have assumed that folk-dance training will support healthy aging more effectively than other types of training—by improving physical performance, blood pressure and glucose metabolism. Moreover, we hypothesized that the positive effect of physical training will be accompanied by changes in blood concentrations of exerkines.

## Results

### Baseline participant’s characteristics

The group's characteristics are presented in Table 1. All participants reported low daily physical activity levels measured by The Physical Activity Questionnaire—IPAQ (short version). There were significant baseline differences between BG and CG groups in the following parameters: height, fat-free mass (FFM), skeletal muscle mass (SMM), total cholesterol (TC),and low-density lipoprotein (LDL-C). The applied training intervention did not cause any change in the body composition.

**Table 1:** Baseline and post-training values of anthropometric parameters, lipid profile, blood pressure, and functional fitness.

Variables	BG (n = 15)			DG (n = 14)			CG (n = 12)		
	I	II	p*	I	II	p*	I	II	p*
Anthropometric parameters									
Weight (kg)	73.6 ± 15.0	73.3 ± 15.3	0.997	84.5 ± 22.6	83.0 ± 21.6	0.522	88.1 ± 13.5	87.7 ± 13.8	0.499
BMI (kg/m^2^)	27.5 ± 4.0	27.8 ± 4.4	0.958	30.4 ± 6.4	29.9 ± 6.0	0.186	29.6 ± 3.7	29.5 ± 4.0	0.999
FFM (kg)	49.3 ± 10.4^#^	49.6 ± 10.4	0.976	52.7 ± 12.3	51.9 ± 11.8	0.999	60.3 ± 10.3	59.3 ± 9.7	0.584
SMM (kg)	27.0 ± 6.1^#^	27.3 ± 6.3	0.960	29.2 ± 7.4	29.3 ± 7.0	0.999	33.3 ± 6.0	33.0 ± 5.6	0.708
BFM (kg)	24.3 ± 9.2	24.0 ± 9.7	0.998	31.7 ± 13.6	30.8 ± 13.5	0.162	27.8 ± 8.5	28.7 ± 8.9	0.741
VFA (cm^3^)	103.0 ± 34.8	97.2 ± 33.2	0.998	129.4 ± 46.1	118.8 ± 42.9	0.053	124.6 ± 32.0	126.5 ± 34.3	0.999
Lipid profile									
TC (mg/dL)	210.1 ± 31.7^#^	199.9 ± 37.4	0.555	183.9 ± 28.5	168.3 ± 22.4	0.164	171.0 ± 31.4	174.0 ± 41.7	0.997
TG (mg/dL)	109.9 ± 48.9	105.8 ± 58.7	0.997	116.8 ± 45.5	116.3 ± 31.2	0.999	110.8 ± 43.6	118.4 ± 51.6	0.969
LDL-C (mg/dL)	125.5 ± 28.1^#^	124.9 ± 60.6	0.998	103.5 ± 25.6	91.4 ± 18.4	0.881	94.4 ± 25.2	99.6 ± 27.9	0.997
HDL-C (mg/dL)	60.0 ± 12.7	64.8 ± 23.2	0.682	55.9 ± 7.0	54.7 ± 8.7	0.999	53.7 ± 12.0	56.3 ± 14.0	0.977
Blood pressure									
SBP (mmHg)	147.2 ± 9.9	133.9 ± 17.6	**0.001**	146.8 ± 7.6	133.8 ± 5.5	**0.003**	143.3 ± 8.8	144.2 ± 10.8	0.999
DBP (mmHg)	80.0 ± 9.4	70.2 ± 10.2	**0.001**	78.0 ± 2.7	72.0 ± 6.4	0.079	75.1 ± 6.9	75.3 ± 6.4	0.999
Functional fitness									
TUG (s)	7.1 ± 0.9	6.1 ± 1.0^Δ^	**0.001**	6.9 ± 0.7	5.9 ± 0.6^Δ^	**0.003**	7.4 ± 1.3	8.1 ± 1.8	0.087
6MWT (m)	359.5 ± 35.8	424.5 ± 43.8^Δ^	**0.001**	369.9 ± 16.7	434.1 ± 50.6^Δ^	**0.001**	357.4 ± 62.5	362.0 ± 69.4	0.997

Values are given as mean ± SD.

*BG* balance training group, *DG* dance training group, *CG* control group, *I* values at baseline, *II* values post training, *BMI* body mass index, *FFM* free fat mass, *SMM* skeletal muscle mass, *BFM* body fat mass, *VFA* visceral fat area, *TC* total serum cholesterol, *TG *triglycerides, *LDL-C* low-density lipoprotein cholesterol, *HDL-C* high-density lipoprotein cholesterol, *SBP* systolic blood pressure, *DBP* diastolic blood pressure, *TUG* time up and go test, *6MWT* 6-min walk test.

Significant values are in bold.

*p—p-value applying two-way repeated-measures ANOVA before and after intervention.

^#^p < 0.05, a significant difference at baseline vs. CG.

^Δ^p < 0.05, a significant difference after training vs. CG.

### Changes in physical performance after 12 weeks of training

The 12-week training programs positively impacted participants' physical performance, functional balance, and mobility. Women in both BG and DG groups showed significant improvement in the distance covered during the 6-min walking test (Table 1), whereas no significant differences in the evaluated parameter were noted in the CG. The distance increased from 359.5 ± 35.8 to 424.5 ± 43.8 m in the BG (p = 0.0001) and from 369.9 ± 16.7 to 434.1 ± 50.6 m in the DG (p = 0.0001). The final differences between BG and CG (p = 0.020) and between DG and CG (p = 0.005) were statistically significant. The time in the TUG test significantly decreased in both BG (from 7.1 ± 0.9 to 6.1 ± 1.0 s; p = 0.0006) and the DG (from 6.9 ± 0.7 to 5.9 ± 0.6 s; p = 0.0039) groups post-intervention (Table 1). There were significant differences between BG and CG (p = 0.0005) and between DG and CG (p = 0.0002) at the end of the experiment. No significant changes were observed in the CG after 12 weeks of intervention.

### Alteration in glucose homeostasis markers after 12 weeks of interventions

**Figure 1:**
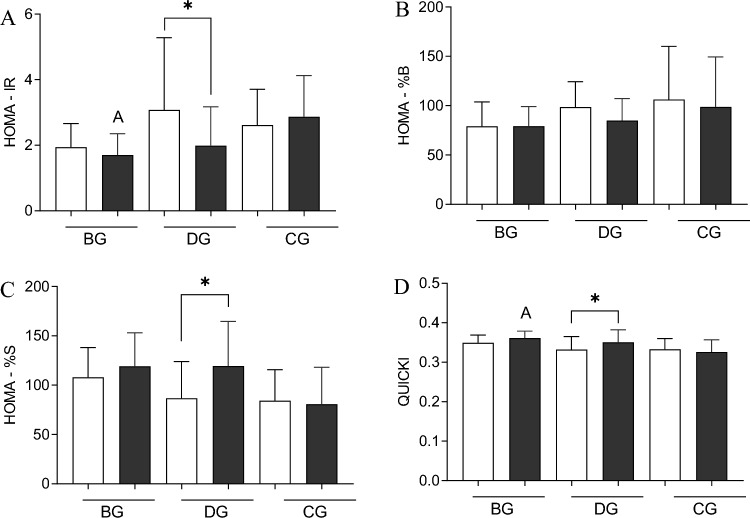
Post-training changes in insulin sensitivity and resistance indicators. (**A**) HOMA-IR; (**B**) HOMA-%B; (**C**) HOMA-%S; (**D**) QUICKI. There were significant differences between the indicated time points: *p < 0.05; between the groups: A—< 0.05 after training vs. CG. The data are presented as the means ± SEM; white before and black color after the intervention.

The 12-week training programs improved glucose metabolism and insulin sensitivity in the cohort of elderly (Fig. 1). The most favorable changes were observed in response to dance training. In the DG group HOMA-IR was reduced from 3.1 ± 2.3 to 2.0 ± 1.2 (p = 0.023), HOMA-%S and QUICKI significantly increased from 86.8 ± 38.7 to 119.4 ± 47.1 (p = 0.020), and 0.33 ± 0.04 to 0.35 ± 0.03 (p = 0.035), respectively (Fig. 1A, C, D). In the BG, there were similar tendencies in HOMA-IR reduction (from 1.9 ± 0.8 to 1.7 ± 0.7) and HOMA-%S and QUCKI improvement (107.9 ± 31.3 to 119.0 ± 35.4, and 0.35 ± 0.02 to 0.36 ± 0.02, respectively) but these changes were not statistically significant. Further, the QUICKI index significantly differed between BG and CG after the intervention (p = 0.034). A slight decline in insulin level was noted in both training groups (from 7.6 ± 2.4 to 7.0 ± 2.0 µIU/ml in the BG and from 9.5 ± 3.2 to 7.4 ± 2.7 µIU/ml in the DG) (Fig. 1A). However, the post-intervention values significantly differed between BD and CG (p = 0.019) and between DG and CD (p = 0.039). After 12 weeks of training, no changes in glucose and insulin concentrations were noted (Figure S1).

### Changes in lipid metabolism and inflammatory status after 12 weeks of training

**Figure 2:**
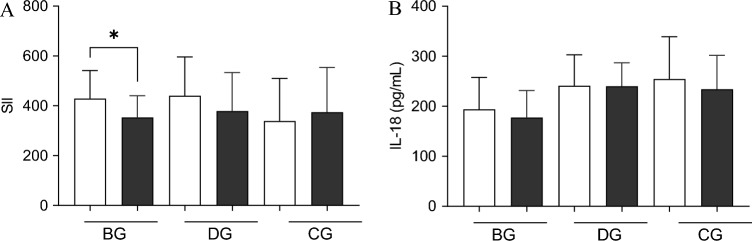
Post-training changes in inflammatory status. (**A**) SII index; (**B**) interleukin-18. There were significant differences between the indicated time points: *p < 0.05. The data are presented as the means ± SEM; white before and black color after the intervention.

The intervention induced a slight but insignificant reduction of TC concentration in both training groups (from 210.1 ± 31.7 to 199.9 ± 37.4 mg/dl in the BG and from 183.9 ± 28.5 to 168.3 ± 22.4 mg/dl in the DG) (Table 1). No changes were observed in the CG. After 12 weeks of dance training, but not balance training, a tendency to decrease in LDL-C was recorded (from 103.5 ± 25.6 to 91.4 ± 18.4 mg/dl). Any changes were also observed in HDL-C and triglycerides in each group participating in the experiment. Still, we recorded the significant amelioration of SII after 12 weeks of training in the BG (from 429.1 ± 116.7 to 353.3 ± 90.4; p = 0.017), and the same tendency in the DG group (from 440.5 ± 156.2 to 379.0 ± 154.3) (Fig. 2A). No effect of either group or time was noted on IL-18 concentration (Fig. 2B). 25-(OH)D concentration were homogenous among the groups (23.8 ± 10.8 – 29.0 ± 15.3 ng/ml).

### The effect of the 12 weeks of a training procedure on blood pressure

The training programs significantly improved systolic (SBP) and diastolic blood pressure (DBP) (Table 1). SBP was significantly reduced in BG (from 147.2 ± 9.9 to 133.9 ± 17.6 mmHg; p = 0.0003), as well as in the DG (from 146.8 ± 7.6 to 133.8 ± 5.5 mmHg; p = 0.0035). In the BG, also a significant reduction in DBP was noted (from 80.0 ± 9.4 to 70.2 ± 10.2 mmHg; p = 0.0002), and the same tendency was noticed in the DG (from 78.0 ± 2.7 to 72.0 ± 6.4 mmHg). No changes in both SBP or DBP were recorded in the CG group.

### Exerkines profile at baseline and post the 12 weeks of training

**Figure 3:**
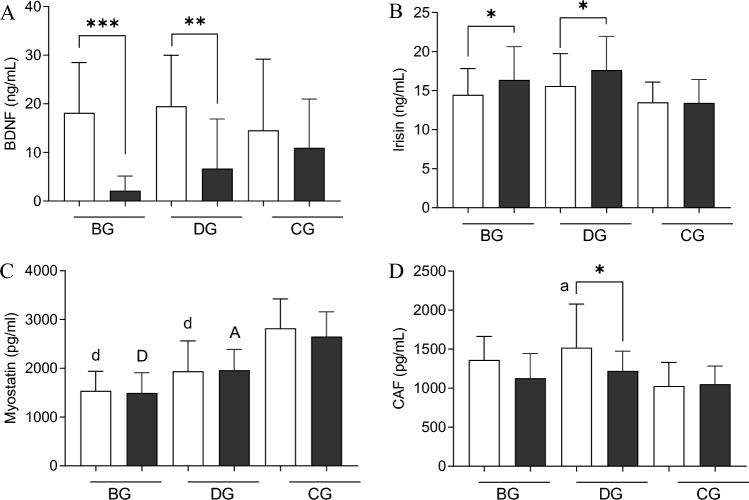
Post-training changes in exerkines concentrations. (**A**) BDNF; (**B**) irisin; (**C**) myostatin; (**D**) CAF. There were significant differences between the indicated time points: *p < 0.05, **p < 0.01, ***p < 0.001; between the groups: a—p < 0.05 before training, A—< 0.05 after training; d—p < 0.001 before training; D—p < 0.001 after training vs. CG. The data are presented as the means ± SEM; white before and black color after the intervention.

BDNF concentration significantly decreased in both BG (from 18.1 ± 10.7 to 2.1 ± 3.1 ng/ml; p = 0.0002) and DG (from 19.5 ± 10.9 to 6.7 ± 10.6 ng/ml; p = 0.0025), while it did not change in the CG (Fig. 3A). Irisin concentration significantly increased in both training groups (from 14.5 ± 3.5 to 16.4 ± 4.4 ng/ml; p = 0.029 for BG, and from 15.6 ± 4.3 to 17.6 ± 4.5 ng/ml; p = 0.022 for DG), whereas its concentration remained unchanged in the CG group (Fig. 3B). The training interventions did not induce changes in myostatin concentration (Fig. 3C). Significant changes were, instead, observed in CAF. In the DG, the significant reduction in CAF concentration was noted after training (from 1519.3 ± 581.6 to 1221.3 ± 262.2 pg/ml; p = 0.024), whereas, in the BG, only the same tendency was recorded (from 1362.5 ± 313.1 to 1127.5 ± 329.9 pg/ml; p = 0.073). Moreover, at the end of the intervention, CAF levels differed between DG and CG (p = 0.026) (Fig. 3D).

## Discussion

The main finding of the current study is that a 12-week folk-dance training performed by older adults significantly improved insulin sensitivity indicators with a significant drop in BDNF and the rise of irisin concentrations. Additionally, CAF, a potential marker for muscle atrophy, resulted in a reduced association with improved physical performance. It is worth to note that such beneficial effects in insulin sensitivity were pronounced, especially among those subjects who presented the worse condition (i.e., frank insulin resistance) at baseline.

The improvement in insulin sensitivity and resistance indicators was visible mainly in HOMA-IR, HOMA-%S, and QUICKI. The positive effects were more pronounced in the folk-dance training group compared to the balance training group, among whom only a tendency to improve insulin sensitivity indicators was observed. The discrepancies between groups may result from slight, although not significant, differences in baseline values of these parameters. These results reflect the findings of previously published research, where the positive effect of physical training was more pronounced among people with type 2 diabetes, hypertension, hyperlipidemia, or metabolic syndrome. Indeed, the recorded reductions in total cholesterol, LDL, fasting insulin, and HOMA-IR were not as pronounced in healthy subjects^20^. Further, the latest studies indicated that the cut-off point of HOMA-IR for predicting the prevalence of metabolic syndrome is 1.8, and a value of 1.62 was obtained for identifying individuals at risk of IR^30,31^. Both training groups in the current study are above this cut-off point. Thus, we could summarize that moderate-intensity aerobic exercise effectively counteracts risk factors for CVD and glucose metabolism indexes.

Together with beneficial changes in insulin sensitivity registered in our trained groups, we noted the reduction of blood pressure. Although the awareness of the need for physical activity is growing, the elderly may be afraid of undertaking regular physical activity. Both resistance and long-term aerobic training have been commonly suggested to reduce blood pressure in middle-aged individuals with pre-hypertension and frank hypertension^32^. The magnitude of blood pressure reduction observed after physical training might be comparable to those induced by first-line anti-hypertensive drugs^33^. Our results supported these observations and proved that both moderate-intensity training programs effectively reduce blood pressure. The potential mechanism underlying this effect may be the aerobic exercise-induced increases in nitric oxide (NO) bioavailability, consequently improving endothelial function and endothelium-dependent vasodilation^32^.

Additionally, we hypothesize that the amelioration in insulin resistance was associated with a reduction in BDNF concentrations.The primary function of BDNF is to regulate neurogenesis in the brain; however, it has also been shown to have a pivotal role in the regulation of peripheral metabolism, especially energy balance and insulin sensitivity^34^. Although the mechanism of action of BDNF in the central nervous systems quite well known, the peripheral pathways are not well understood. A novel metabolic pathway has been presented by Fulgenzi et al. It has been proposed in a mouse model that the BDNF receptor TrkB.T1 is expressed by pancreatic β-cells where it regulates insulin release^14^. Previous human research has shown that low circulating concentrations of BDNF are associated with insulin resistance, type 2 diabetes, and cognitive impairments^12^. However, Boyuk and co-workers have also observed contradictory results, who found higher serum BDNF concentration in T2M patients and a positive correlation with HOMA-IR and triglycerides^35^. It has been suggested that BDNF, through crossing the blood–brain barrier, may shift to the central nervous system and ameliorate the detrimental effects of insulin resistance in the brain as an antioxidant and neurotrophic factor^36,37^.

The results from studies conducted on humans are ambiguous regarding the relationship between BDNF and long-term exercise protocols. No effects^38^, enhancement in circulating BNDF concentration^15,39^, and decrease have been observed^40^.Previously published studies indicated that the post-exercise increase in BDNF concentration was associated with improved mood, cognitive functions, and quality of life^41,42^. However, the elevation of BDNF and improvement of cognitive functions depended on the subjects' age, the intensity of exercise, and metabolic factors, including, for instance, peripheral lactate concentration, insulin-like growth factor-1 (IGF-1), and vascular endothelial growth factor levels (VEGF)^43^. When considering the high-intensity exercise modality, most of these observations were about young, healthy subjects. In agreement with previous results^40^, our study indicates that serum BDNF concentration decreased after aerobic training, regardless of the type, associated with the reduction in HOMA-IR and the increase in QUICKI indexes.

Another exerkine that may regulate insulin resistance in response to physical training is irisin. Irisin is expressed in skeletal muscle and other tissues and seems to induce a brown-like phenotype in some white adipocytes, which improves multiple metabolic parameters by increasing energy expenditure^44^. Therefore, irisin could potentially protect against different conditions such as cardiovascular diseases, type 2 diabetes mellitus, or fatty liver disease. In the current study, we observed an amelioration in insulin resistance, especially in the dance training group, with a positive association with circulating irisin, supporting the hypothesis of an insulin-like action of irisin. Irisin, indeed, enhances the expression of genes involved in glucose transport and lipid metabolism in myocytes (GLUT4, HK2, and PPARA), inhibits the expression of genes that are involved in glycogenolysis or gluconeogenesis and downregulates proteins associated with insulin resistance pathway^45,46^.

Along with insulin sensitivity improvement, the reduction of the systemic immune-inflammation index was evidenced in the balance group and a trend to reduction in the folk-dance group. In the last published review, authors pointed out that irisin-mediated alterations in cytokine production results in reduced macrophage migration and infiltration, vascular leukocyte adhesion and migration, and acute phase inflammatory response^47^. Thus, it cannot be ruled out that the drop in SII was the effect of irisin's action. The reduction of the SII index and changes in irisin have particular meaning due to the potential role of irisin in obesity-related cancer prevention as well as in osteoporosis and neurodegenerative diseases- commonly noted among aged people^48^. Noteworthy, sarcopenia's pathophysiological mechanism(s) contemplates the deterioration of the homeostatic systems, including the immune system. In opposition to the previous observation, any alternations were recorded in other pro-inflammatory cytokine IL-18^49^ in the current study. Also, Gomarasca et al. did not indicate changes in circulating IL-18 concentrations in obese men and women following a 12-week moderate-intensity aerobic NW training program^50^. However, among our subjects at baseline, a significant positive correlation was noted between IL-18 and visceral fat area, insulin, HOMA-IR, and triglycerides, and a negative correlation with HOMA-%S and QUICKI index. These results suggest that IL-18 could take part in the pathogenesis of obesity and insulin resistance, but it may not be sensitive to exercise intervention.

Although we did not determine muscle mass, our study focused on motor and functional balance test results (TUG and 6MWT). In both training groups, together with shifts of BDNF and irisin, the amelioration of these muscle functions was recorded. As irisin has been suggested as muscle wasting and muscular performance biomarker^23^, these changes are significant since sarcopenia (the decline of fibers numbers and its size reduction) and dynapenia (reduction of strength) are highly prevalent in the elderly^51^. The scientific data point out that dynapenia precedes the process of sarcopenia, which is expressed by weakening motor abilities. Thus, the attenuation of this drop and the improvement of glucose homeostasis can be considered a preventive strategy against sarcopenia. Therefore, changes in these two proteins may indicate a positive effect of this training on preventing age-related loss of muscle mass.

Among the proteins considered as biomarkers of muscle atrophy and sarcopenia analyzed in this study—myostatin and CAF, only the latter substantially changed. Although previous studies showed a reduction in myostatin concentration, we did not observe such an effect. However, different results have been reported depending on sex, age, and exercise type (aerobic or resistance)^52^. The myostatin decrease was mainly observed after resistance forms of training^53^. Significant post-training changes have been noted in CAF—one of the best indicators of neuromuscular junction integrity and thus may be a potential biomarker for the progression of age-related functional decline and muscle atrophy^20^.CAF is associated with one of the mechanisms of functional deterioration in aging—a decline in neuromuscular junction integrity. This pathology leads to sarcopenia development and worsening in physical performance^24^. The results of our study suggest that dance training may be effective in coping with functional decline related to aging via the reduction of CAF concentration. Only a few studies assess changes in circulating CAF levels following physical exercise^20,25^. It should be indicated that previous researches show both increases, no effects, and decrease in circulating CAF concentration after exercise interventions. One of the previous researches indicated that one year of physical activity intervention combining moderate-intensity walking, strength, and balance exercises did not reduce CAF concentration^20^. On the other hand, Fragala and co-workers observed that a 6-week resistance training increased CAF concentration in a group of elderly^25^. However, this study assessed the resistance form of training. One study confirmed our observations and indicated that recreationally active aged individuals, who regularly practiced dance for more than 3 years, had significantly lower CAF concentration compared to their counterparts involved in other aerobic physical activities. Additionally, elderly dancers demonstrated better dynamic balance and functional performance^26^. In the current study, a significant decrease in CAF concentration was observed in the dance training group, with an improvement in dynamic balance and physical performance, measured by TUG and 6MWT tests. Somehow, in the balance training group, a tendency to reduction in circulating CAF was also observed; however, this result was not statistically significant. The obtained results suggested that dance training is more effective in protecting from NMJ degeneration. We are far from speculating, but dance training may induce greater neuroplasticity and NMJ health than resistance or aerobic training since it involves both motor and sensory, cognitive and coordinative^54^. Moreover, dance training belongs to creative activities.

This study presents some limitations that should be highlighted. Future studies should include assessing cognitive functions to evaluate if the changes in exerkines are related to cognition improvement. However, for this purpose, previous studies, including ours, indicated that dance training is an effective method to improve cognitive abilities^29^ due to the engagement in sensory, cognitive, and coordinative functions^54^.

Overall obtained data indicated that both training programs effectively improve physical performance, dynamic balance, and inflammatory status and reduce high blood pressure. However, folk dance appears to be more beneficial in ameliorating insulin resistance. The changes in response to training interventions are connected with shifts in BDNF, irisin, and CAF concentrations. Since there is limited data regarding the direct connection between BDNF, irisin, and CAF in humans, obtained results have particular meaning and fill the gaps in this research area.

## Materials and methods

### Participants

**Figure 4:**
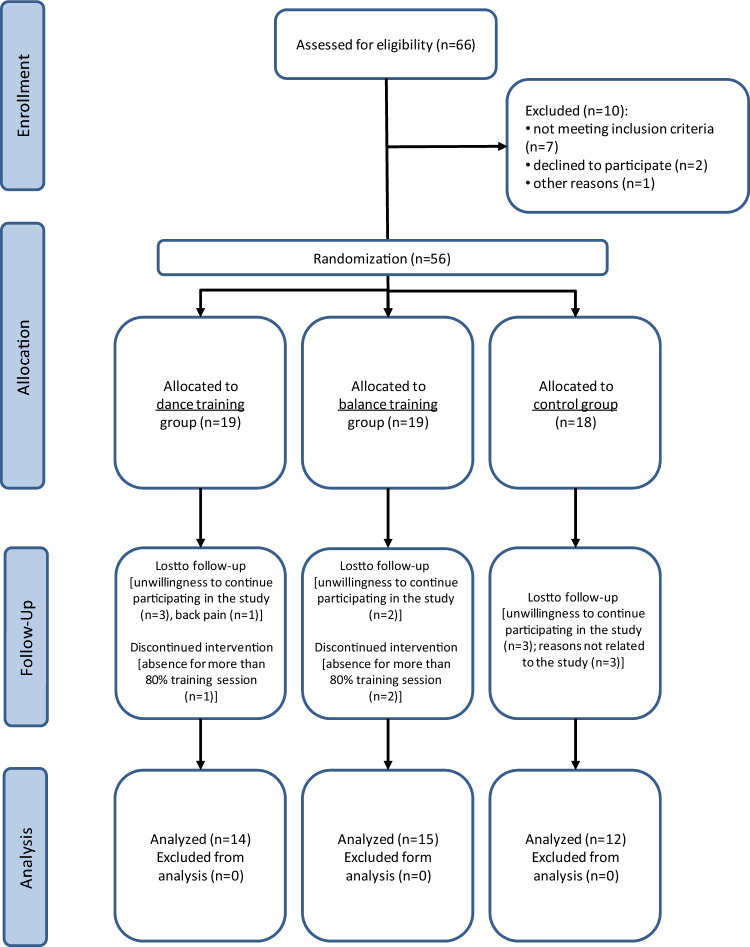
CONSORT flow diagram of the study.

Fifty-six community-dwelling older female (n = 30) and male (n = 26) participants (age 71.3 ± 5.5 years, range 65–85) were enrolled in the study (Fig. 4). Participants were recruited from advertisements in community centres in Gdansk, Poland. All participants lived and functioned independently without any severe cognitive or physical impairments. The inclusion criteria for the study were age ≥ 65 years and low-to-moderate physical activity assessed by the International Physical Activity Questionnaire-IPAQ (short version). The exclusion criteria were any diseases accompanied by contraindication to exercise and require specialized treatment, coronary disease, type 1 or 2 diabetes, grade II or III obesity (measured by BMI), arrhythmia, implanted pacemaker, heart failure, depression, cancer, significant orthopaedic injuries. Participants who take medications for depression, osteoporosis, heart diseases, lipid-lowering, or estrogenic replacement drugs were also excluded and those who regularly participated in exercise programs for 12 months before the study. During the follow-up, 15 of the 56 participants withdrew from the study. Forty-one older adults (21 females and 20 males) completed the study. All participants were informed about the risks and study purpose and were familiarized with the research methods. Written informed consent was obtained from the participants of the study. The study was conducted in accordance with the Declaration of Helsinki. The experiment was approved by the Bioethical Committee of the Regional Medical Society in Gdansk (KB-34/18).

### Study design

Participants were randomly assigned into three groups: balance training group (BG; n = 15), dance training group (DG; n = 14), and control group (CG; n = 12). The BG and the DG groups performed training three times a week (Monday, Wednesday, and Friday) for 12 weeks. All measurements (body composition analysis, blood pressure measurement, blood collection, and functional tests—6-min walk test (6MWT) and timed up and go test (TUG) were performed 2 days before and 2 days after the intervention. Blood samples were taken to determine blood irisin, myostatin, IL-18, BDNF, CAF, 25-(OH)D3, insulin, and glucose concentration. Before the beginning of the training protocols, a medical examination was performed to exclude those participants with significant contraindications to exercise. The physician also assayed the blood pressure (BP) before and after the intervention. BP was measured in the seated position, on the left arm, thrice with 2-min intervals by the auscultator technique using a conventional mercury sphygmomanometer with an appropriately sized cuff. Measurements were obtained in the morning between 8:00 AM and 10.00 AM, after a 5-min of rest.

The fitness tests were performed by specialists who were blinded to the group assignments, exercise program, and control group status of the participants.

### Body composition analysis

Body composition analysis was estimated by a precise multi-frequency impedance body composition analyzer (InBody 720, Biospace, Korea), using six different frequencies (1 kHz, 5 kHz, 50 kHz, 250 kHz, 500 kHz, and 1000 kHz) at each of five segments of the body (Right Arm, Left Arm, Trunk, Right Leg, Left Leg). The measurement was performed with the 8-point tactile electrode method. InBody assesses parameters of body composition as follows: body weight, free fat mass (FFM), body fat mass (FM), skeletal muscle mass (SMM), and visceral fat (VFA). The body composition analysis was performed by one researcher for all participants in a fasted state, 12 h after the last meal and drink, between 9:00 and 10:00 in the morning after blood collection)^55^.

### Blood collection

Blood samples for irisin, myostatin, IL-18, BDNF, CAF, 25-(OH)D3, insulin, and glucose assessment were collected between 7:00 and 9:00 AM after overnight fasting at two time-points: at baseline and after completion of the 12-week intervention. A qualified nurse took venous blood from the antecubital vein into vacutainer tubes (Vacutainer SSTTM II Advance) for serum separations and tubes with EDTA as an anticoagulant for plasma separation. Samples were centrifuged at 2000*g* for 10 min at 4 °C. The separated plasma and serum samples were frozen and stored at − 80 °C until later analysis.

### Biochemical assays and insulin sensitivity indicators

Serum glucose was measured by the colorimetric enzymatic method (Randox manual gl2623). Serum insulin concentration was assessed by the immunoezymatic (ELISA) method (Demeditec diagnostics, Germany, no. DE2935). The maximal intra-assay coefficient of variability (CV) was 2.6–1.8%, and the inter-assay CV was 2.9–6.0%. The assay sensitivity was 1.76 µIU/ml. The following formulas calculated insulin sensitivity according to QUICKI and HOMA-IR: QUICKI = 1/(log(fasting insulin [µU/ml] + log(fasting glucose [mg/dl])), HOMA-IR = (fasting serum insulin µU/ml × fasting plasma glucose mmol/l/22.5). The updated computer HOMA2 model was used to determine the β-cell function (HOMA-%B) and insulin sensitivity (HOMA-%S) from paired fasting glucose and insulin level. The HOMA-indexes were obtained by the software HOMA 2 Calculator, version 2.2.3, copyrighted by The University of Oxford. In order to assess if the inflammation status has modified exercise-induced response, the systemic immune-inflammation index (SII) was calculated based on the formula proposed by Chen and co-workers: SII = (P × N)/L, where P, N, and L refer to peripheral platelet, neutrophil, and lymphocyte counts^56^.

### Exerkines blood concentration

Irisin and myostatin concentrations were determined by immunoenzymatic (ELISA) method using commercially available kits(Phoenix Pharmaceuticals Inc, no. EK067-29 for irisin, and R&D, United States&Canada, no. DGDF80 for myostatin) according to the manufacturer's protocol. The maximal intra-assay CV was 4–6%, and inter-assay CV was 8–10% for irisin and 1.8–5.4%, and 3.1–6.0% for myostatin. The assay sensitivity was 1.29 ng/ml for irisin and 5.32 pg/ml for myostatin. BDNF was assessed in serum using ELISA Kit (R&D, United States&Canada, no. DBNT00). The maximal intra-assay CV was 3.2–3%, and inter-assay CV was 7.2–4.7% for BDNF, and the assay sensitivity was 1.35 pg/ml.IL-18 was assessed in serum using ELISA Kit (R&D, United States&Canada, No. QK318). The maximal intra-assay and inter-assay CV for IL-18 were 2.5–4% and 5.4–7.9%, respectively, and the assay sensitivity was 4.57 pg/ml. Serum CAF was determined using Fine Test ELISA Kit—no. EH4820. The assay sensitivity was 28.125 pg/ml for CAF. The maximal intra-assay CV was < 8%, and inter-assay was < 10% for CAF. According to the manufacturer's protocol, serum 25(OH)D3 was assessed by ELISA Kit (Demeditec diagnostics, Germany, No. DE1971). The maximal intra-assay CV was 2.5–7.8%, and the inter-assay was 7.4–9.2%. The assay sensitivity was 2.81 ng/ml. The concentration of 25-(OH)D3 below 30 ng/ml was classified as insufficient.

### Physical performance assessment

The time up and go test (TUG) test assessed functional balance and mobility and identified potential fallers. It was shown that a test time of 13.5 s or above is associated with a two- to three-fold higher risk of falls^57^. 6-min walk test (6MWT)assessed the walking distance of patients within 6 min. The test result could indicate the functional status and elders' cardiovascular and locomotor systems^58^. The functional tests were performed as described previously^59^.

### Balance training protocol

Two days after the completion of baseline measurements, women from the BG participated in the training program. The training was conducted three times a week for 12 weeks, with 36 training units as described previously^29^. Each training session lasted 50 min, including 10 min warm-up, 30 min balance training, and 10 min stretching and respiratory activity. Each training session was performed under the supervision of a physiotherapist (Master's degree in physiotherapy, specializing in conducting exercise programs with older adults). All training sessions were performed for the entire group in the training hall. Only those participants who attended at least 80% of training sessions were qualified for statistical analysis.

### Dance training protocol

Two days after baseline measurements, women from the DG group participated in the dance training program as described in our previous study^29^. Participants assigned to the DG performed 50 min of dance training to Polish folk music. The training was performed in the group in a training hall and was conducted three times a week for 12 weeks. Each training session lasted for 50 min and included 10 min warm-up, 30 min of folk-dance training. Each exercise session was performed under the supervision of a qualified dance coach. The same rules of participants' attendance were concerned.

### Control group

The control group did not participate in any training intervention. Participants were instructed not to change their daily habits during the intervention phase.

### Statistical analysis

Statistical analyses were performed using the Statistica v.13 software package. The results are expressed as the mean ± standard deviation (SD). The Shapiro–Wilk and Brown-Forsyth tests were performed to test the normality of parameter distribution and group variances equality. For data with normal distribution and equal variations, the baseline differences between groups were tested using the one-way ANOVA model. For data without normal distribution and equal variations, the non-parametric Kruskal–Wallis test was used to test between-group differences at baseline. The changes in mean scores were tested using the two-way repeated-measures ANOVA models; if a difference was detected in the ANOVA models, the significant differences were determined using Tukey's post-hoc test. The results were considered statistically significant when p ≤ 0.05. A Pearson product-moment correlation coefficient was computed to assess the relationship between the obtained results.

## Supplementary Information

Supplementary Figure S1.

## Data Availability

The datasets analyzed during the current study are available from the corresponding author upon reasonable request.
