# Supplementary material for: Changes in selected exerkines concentration post folk-dance training are accompanied by glucose homeostasis and physical performance improvement in older adults
Source: Sci Rep. 2023 May 26;13:8596. doi: 10.1038/s41598-023-35583-w (PMC10215059; doi:10.1038/s41598-023-35583-w)
Supplement: Supplementary file 1 — Supplementary Figure S1. [file 41598_2023_35583_MOESM1_ESM.pdf]

Figure S1

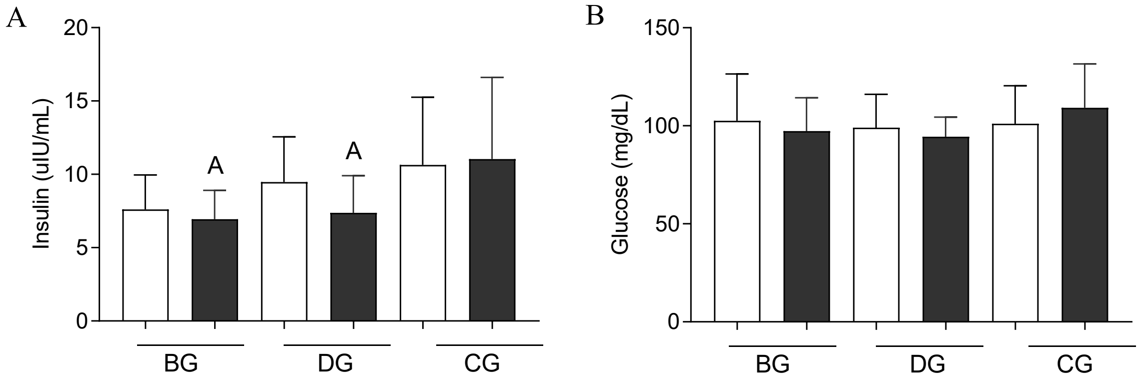

Figure S1. Post-training changes in (**a**) insulin concentration; (**b**) glucose concentration. There were significant differences between the groups: A – <0.05 after training vs. CG. The data are presented as the means ± SEM; white before and black color after the intervention.
